# Using Death Certificate Reports to Find Severe Leptospirosis Cases, Brazil

**DOI:** 10.3201/eid1310.070150

**Published:** 2007-10

**Authors:** Anne Spichler, Daniel Athanazio, Marcia Buzzar, Bronislawa Castro, Erica Chapolla, Antonio Seguro, Joseph M. Vinetz

**Affiliations:** * Health Municipality Secretariat of São Paulo, São Paulo, Brazil; † University of São Paulo School of Medicine, São Paulo, Brazil; ‡ Federal University of Bahia, Salvador, Brazil; § Laboratory Center for Zoonosis Control, São Paulo, Brazil; ¶ University of California San Diego School of Medicine, La Jolla, California, USA

**Keywords:** Leptospirosis, zoonotic disease, notification, emerging infectious disease, Brazil, dispatch

## Abstract

Severe leptospirosis with pulmonary hemorrhage is emerging globally. Measures to control leptospirosis through sanitation depend on accurate case finding and reporting. Rapid death certificate reporting, plus necropsy of persons who died of leptospirosis, facilitates public health intervention and could provide an important tool in assessing the global burden of leptospirosis.

Although pulmonary involvement has been well recognized as a major component of leptospirosis (*1*), worldwide attention to severe pulmonary hemorrhagic syndrome (SPHS) emerged after an outbreak in rural Nicaragua in 1995 in which most cases lacked jaundice and renal failure, the classic manifestations of severe leptospirosis (*2*). A report from Peru, where leptospirosis is endemic, pointed out that SPHS can complicate human leptospirosis without jaundice or renal failure (a condition known as Weil syndrome) (*3*). Although not well recognized globally in general clinical practice, SPHS is considered a major clinical problem in some leptospirosis-endemic regions, ranging from the Andaman and Nicobar Islands (*4*) to urban coastal Brazil (*5*).

Epidemic leptospirosis most commonly occurs after flooding in densely populated centers in developing countries, and it continues to be an important clinical problem in urban coastal Brazil. São Paulo is the most populous Brazilian state (population ≈40 million). The annual reported incidence of leptospirosis from 1969 through 1996 was 0.53–1.13 cases per 100,000 inhabitants (*6*). In the São Paulo metropolitan area (population 10.5 million), which accounts for 68% of all reported cases, the annual incidence of leptospirosis was 1.9–3.7 cases per 100,000 inhabitants in the period 1998–2004. During the same period, case-fatality rates varied from 11.0% to 18.0% (Health Municipality Secretariat of São Paulo, unpub. data). In the São Paulo urban setting, SPHS is a common feature of leptospirosis. A case series of São Paulo patients with leptospiral SPHS found that 23 (55%) of 42 patients died from this disease from 1994 through 1997 (*7*).

## The Study

In 2004, PRO-AIM (Program for Improvement of Death Cause Information in São Paulo, abbreviated from the Portuguese name) was initiated in the São Paulo metropolitan area to provide better reporting of causes of death. Leptospirosis is a major infectious disease in São Paulo, and we started active surveillance for cases of acute renal failure and jaundice, pulmonary hemorrhage, or a combination of both. Detailed questionnaires on clinical and epidemiologic features were completed, and necropsies were performed to improve identification of fatal leptospirosis cases in São Paulo.

The central component of this program is a death certificate system. Fatal cases of leptospirosis are a component of this registry as a major category. Death certificates with any clinical features suggestive of leptospirosis or isolated SPHS are referred to the Municipal Health Secretariat of São Paulo. The Secretariat analyzes and attempts to confirm such cases. Confirmation protocols include laboratory analysis, clinical and epidemiologic assessment, and verification by necropsy of case-patients who had pathologic features characteristic of leptospirosis in lungs, kidneys, and liver. If fatal leptospirosis is confirmed within 72 hours, public health authorities can be alerted. In a setting of high leptospirosis transmission, this step may forestall expansion of an epidemic. Furthermore, the Brazilian experience, as well as that of other countries (*1*), includes a high rate of misdiagnosis of leptospirosis and other clinically indistinguishable acute febrile illnesses such as dengue or scrub typhus fever. Thus, rapid identification and confirmation of fatal leptospirosis are important tools for surveillance, public health interventions, and alerting clinicians.

We describe our initial experience of actively seeking, identifying, and reporting fatal cases of leptospirosis. Using data from January 1, 2004, through August 31, 2006, we used a cross-sectional approach to analyze death certificate data from the São Paulo metropolitan area. Fatal cases were confirmed in the laboratory by serologic or immunohistochemical examination, supplemented by epidemiologic and clinicopathologic evidence. Pathologic criteria were an important adjunct to confirm cases because ≈30% of deaths of patients hospitalized for leptospirosis occur within 24 hours, which may preclude serologic diagnosis because of delayed antibody responses. Pathologic examination can identify constellations of known complications as well as unexpected features. In fulminant infection, histopathologic findings confirm involvement of typical targeted organs such as acute tubular necrosis or interstitial nephritis, acute loss of cohesion of hepatocytes, and pulmonary hemorrhage, which is important because, in leptospirosis-endemic regions, potential leptospirosis patients may have had previous infection and thus preexisting antileptospiral antibodies (*8*). We emphasize, however, that pathologic criteria are only confirmatory if clinical and epidemiologic criteria for infection are fulfilled. In the experience reported here, 5 cases (which were included in the laboratory confirmation group) were confirmed by immunohistochemical examination. Leptospiral antigen detection in postmortem samples is important for confirming the diagnosis (*9*) but is limited by tissue deterioration if there is a prolonged period between death and necropsy.

In 2004, 42 (15%) of 285 cases of reported leptospirosis cases were fatal; in 2005, 28 (11%) of 262 cases were fatal; and from January 1 through June 2006, 31 (19%) of 167 cases were fatal. The Table shows the distribution of fatal cases from January 2004 through August 2006. Of 101 fatal cases, 62 were confirmed by a combination of serologic and immunohistochemical testing; 15 cases were suggested on the basis of clinical, pathologic, and epidemiologic findings; and 24 were suspected on the basis of strong circumstantial clinical and epidemiologic evidence.

## Conclusions

Isolation of leptospires in culture from clinical specimens is the standard for diagnosis but faces logistical obstacles in real-world settings. In our study, only 6 blood samples were collected for culture, and they were uninterpretable because of contamination. Although not definitive for identifying infecting leptospiral serovars, microscopic agglutination test titers suggested that the most frequently reacting serogroups in São Paulo are Icterohemorrhagiae (72%) and Autumnalis (14%).

Of the 101 fatal cases, necropsies were performed for 42. Of these 42 necropsies, 27 cases were confirmed by a combination of necropsy and positive serologic test results. Fifteen fatal cases were suggested by a combination of necropsy findings plus clinical and epidemiologic evidence.

Of all fatal cases confirmed by necropsy, Weil syndrome with concomitant pulmonary hemorrhage was documented in 86% of cases in 2004, in 67% in 2005, and in 69% in 2006. Less common manifestations included Weil syndrome without pulmonary hemorrhage, and isolated pulmonary hemorrhage (Table).

**Table:** Data from death notification reports in the São Paulo, Brazil, metropolitan area, January 2004–August 2006

Data from reports	2004	2005	2006
Total no. reported leptospirosis cases	285	262	167
Annual incidence per 100,000 inhabitants	2.7	2.4	1.7
Fatality rate, %	15	11	18
Total no. deaths	42	28	31
Fatal cases			
Laboratory confirmed, no. (%)	28 (67)	17 (60)	17 (55)
Clinical and epidemiologically assessed, no. (%)	14 (33)	11 (40)	14 (45)
Total no. necropsies (%)	14/42 (33)	15/28 (53)	13/31 (41)
Frequency of concurrent Weil syndrome and pulmonary hemorrhage, no. (%)	12/14 (86)	10/15 (67)	9/13 (69)
Frequency of Weil syndrome without pulmonary hemorrhage, no. (%)	1/14 (7)	4/15 (27)	3/13 (23)
Frequency of pulmonary hemorrhage, no. (%)	1/14 (7)	1/15 (6)	1/13 (8)

The frequency of clinical manifestations of severe pulmonary disease (with or without hemorrhages) was 76% among fatal cases and 26% among nonfatal cases. Importantly, 46% of all patients with severe pulmonary symptoms died, a finding that is consistent with the literature (*1*,*8*).

The system of active death notification we describe can be an important tool to evaluate the emerging complications of leptospirosis and the global extent of disease due to leptospirosis. Nonetheless, this, as well as any system of active death certification notification, has several limitations. A major problem is increasing the number of necropsies, which may be limited by logistics or cultural norms. Increasing the necropsy rate can be aided by prompt response through the death certificate notification system and involvement of public health authorities. Another problem is the lack of serologic and cultural confirmation. Molecular diagnosis would be ideal and could readily be introduced into this system.

The incidence of SPHS seems to have changed elsewhere, e.g., in Salvador, another urban area of Brazil, where the Copenhageni serovar predominates. Pulmonary hemorrhage seems to have newly emerged since the year 2000 (A. I. Ko, pers. comm.). An active surveillance, death certificate–based reporting system represents an important tool that should provide further insights into the natural history and changing clinical manifestations of leptospirosis infection in diverse geographic regions in which the disease is endemic and epidemic. Systematic reporting focused on the most severe cases of leptospirosis has the potential for providing a data-driven basis for ministry-level policy development, institution of public health control measures, and quantitative assessment of the global severity of leptospirosis.

## References

[1] Bharti AR, Nally JE, Ricaldi JN, Matthias MA, Diaz MM, Lovett MA, Leptospirosis: a zoonotic disease of global importance. Lancet Infect Dis. 2003;3:757–71. 10.1016/S1473-3099(03)00830-2 14652202

[2] Trevejo RT, Rigau-Perez JG, Ashford DA, McClure EM, Jarquín-González C, Amador JJ, Epidemic leptospirosis associated with pulmonary hemorrhage-Nicaragua, 1995. J Infect Dis. 1998;178:1457–63. 10.1086/314424 9780268

[3] Segura ER, Ganoza CA, Campos K, Ricaldi JN, Torres S, Silva H, Clinical spectrum of pulmonary involvement in leptospirosis in a region of endemicity, with quantification of leptospiral burden. Clin Infect Dis. 2005;40:343–51. 10.1086/427110 15668855 PMC2366057

[4] Sehgal SC, Murhekar MV, Sugunan AP. Outbreak of leptospirosis with pulmonary involvement in north Andaman. Indian J Med Res. 1995;102:9–12. 7558211

[5] Ko AI, Galvao Reis M, Ribeiro Dourado CM, Johnson WD Jr, Riley LW. Urban epidemic of severe leptospirosis in Brazil. Salvador Leptospirosis Study Group. Lancet. 1999;354:820–5. 10485724 10.1016/s0140-6736(99)80012-9

[6] Romero EC, Bernardo CC, Yasuda PH. Human leptospirosis: a twenty-nine-year serological study in São Paulo, Brazil. Rev Inst Med Trop Sao Paulo. 2003;45:245–8. 10.1590/S0036-46652003000500002 14743663

[7] Marotto PC, Nascimento CM, Eluf-Neto J, Marotto MS, Andrade L, Sztajnbok J, Acute lung injury in leptospirosis: clinical and laboratory features, outcome, and factors associated with mortality. Clin Infect Dis. 1999;29:1561–3. 10.1086/313501 10585813

[8] Levett PN. Leptospirosis. Clin Microbiol Rev. 2001;14:296–326. 10.1128/CMR.14.2.296-326.2001 11292640 PMC88975

[9] Zaki SR, Shieh W-J. Leptospirosis associated with outbreak of acute febrile illnesses and pulmonary haemorrhage, Nicaragua, 1995. Lancet. 1996;347:535–6. 10.1016/S0140-6736(96)91167-8 8596276

